# A Review of the Genetics and Pathogenesis of Syndactyly in Humans and Experimental Animals: A 3-Step Pathway of Pathogenesis

**DOI:** 10.1155/2019/9652649

**Published:** 2019-09-15

**Authors:** Mohammad M. Al-Qattan

**Affiliations:** ^1^Professor of Hand Surgery, King Saud University, Riyadh, Saudi Arabia; ^2^King Faisal Specialist Hospital and Research Center, Riyadh, Saudi Arabia

## Abstract

Embryology of normal web space creation and the genetics of syndactyly in humans and experimental animals are well described in the literature. In this review, the author offers a 3-step pathway of pathogenesis for syndactyly. The first step is initiated either by the overactivation of the WNT canonical pathway or the suppression of the Bone Morphogenetic Protein (BMP) canonical pathway. This leads to an overexpression of Fibroblast Growth Factor 8 (FGF8). The final step is the suppression of retinoic acid in the interdigital mesenchyme leading to suppression of both apoptosis and extracellular matrix (ECM) degradation, resulting in syndactyly.

## 1. Introduction

Studies on developmental biology and embryology of the upper limb have greatly improved our understanding of the genetics and phenotypes of various congenital hand abnormalities [1].

The embryology of normal web space creation [2] and the genetics of human syndactyly [3] are well described in the literature. In this review, the author offers a 3-step pathway of pathogenesis for syndactyly. Based on the review, a classification of syndactyly will be offered based on these steps of pathogenesis.

## 2. Classification of Syndactyly

The genetics literature classifies syndactyly into nine types [3]. However, several other syndromic and nonsyndromic types of syndactyly are not included in this 9-type classification system. Examples include the Saudi-type familial syndactyly which is syntenic to the hammertoe locus in mice [4], the Cenani–Lenz syndactyly phenotype caused by APC mutations [5, 6], the brain atrophy-syndactyly syndrome caused by missense mutations of *FIBULIN1* (OMIM 608180) [7], the triphalangeal thumb polysyndactyly syndrome caused by genomic duplications of the Sonic Hedgehog (SHH) enhancer ZRS (OMIM 174500) [8], acrocephalo-syndactyly syndromes, Greig syndrome (OMIM 175700), and other syndactyly phenotypes associated with GLI3 mutations [9].

## 3. The Unified Pathway of Pathogenesis of Syndactyly

**Figure 1:**
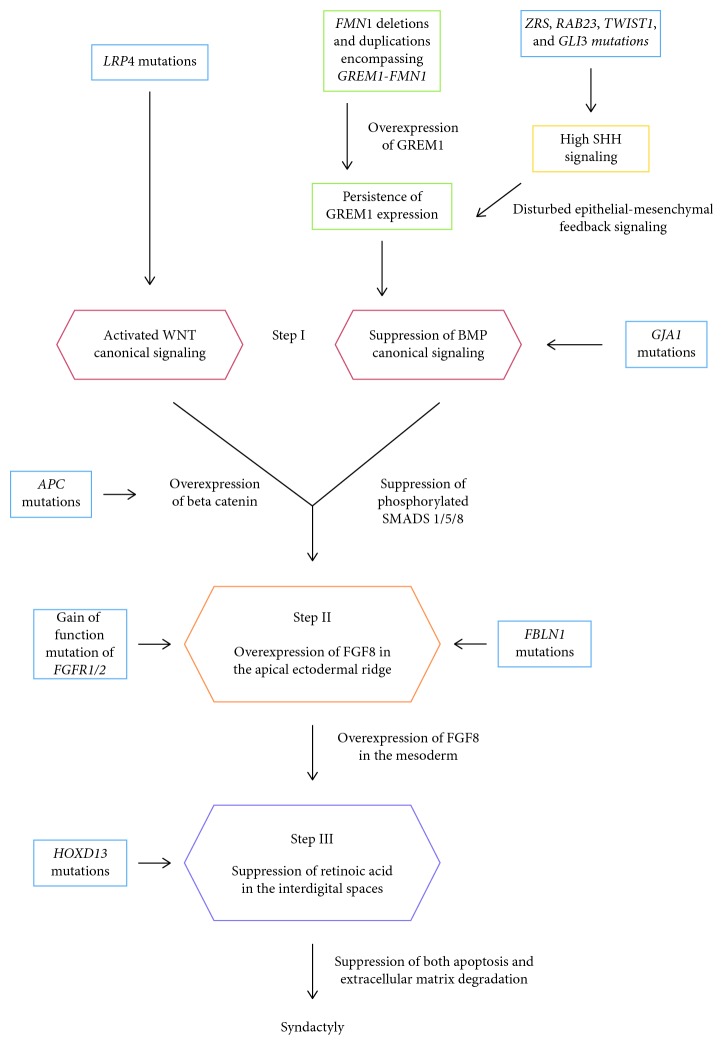
Human syndactyly and the 3-step pathway of pathogenesis.

A 3-step pathway could explain the pathogenesis of almost all types of syndactyly in humans and experimental animals (Figure 1). The first step is either the activation of the WNT canonical signaling or the suppression of the Bone Morphogenetic Protein (BMP) canonical signaling. This will lead to an overexpression of Fibroblast Growth Factor 8 (FGF8) in the apical ectodermal ridge (AER) and also in the mesoderm since FGF8 is a diffusible morphogen [10]. This will then lead to the third step which is the suppression of retinoic acid in the interdigital spaces [11]. FGF8-retinoic acid interactions are mediated through the ERK/MAPK pathway [12, 13]. The suppression of retinoic acid within the interdigital spaces will finally lead to syndactyly because of the suppression of both apoptosis and extracellular matrix (ECM) degradation. Normally, apoptosis is initiated by the action of proapoptotic proteins such as BAX and BAK on the mitochondria [14]. The promotor region of BAX and BAK contains retinoic acid responsive elements [15]. The normal disintegration of the ECM of the web space is mainly mediated by the ADAMTS family of peptidases and MMP11 [16, 17]. The promotor region of the *MMP11* gene also contains retinoic acid responsive elements [18].  Table 1 demonstrates that human syndactyly may be classified according to the 3 steps shown in Figure 1.

### 3.1. Step I: Related Syndactyly in Humans

LRP4 is a strong suppressor of WNT signaling, and hence, loss-of-function mutations of *LRP4* will lead to signal activation and syndactyly (Cenani–Lenz syndrome, OMIM 212780) [19]. Loss-of-function mutations of the *APC* gene lead to beta-catenin accumulation. In most cases, a familial adenomatous polyposis phenotype (OMIM 175100) is seen. However, *APC* mutations may occasionally result in a Cenani–Lenz syndactyly phenotype [5, 6]. Similar Cenani–Lenz phenotypes may also be caused by the overexpression of GREM1 (which is a strong suppressor of the BMP signaling) secondary to *FMN1* deletions or duplications encompassing the *GREM1*-*FMN1* genes [20].

The normal events of the epithelial-mesenchymal feedback loop [21] participate in the process of normal web separation which occurs in humans between the 19^th^ and the 22^nd^ embryonic stages (47–54 intrauterine days) [22]. Prior to stage 19, the digits are normally webbed. During this time, the high SHH activity stimulates the expression of GREM1, maintaining a high level of FGF8 and a low level of retinoic acid, and the digits are maintained in the webbed state. The growth of hand paddle during stages 19–22 leads to an increase in the gap between the SHH signal and GREM1-expressing cells. Hence, SHH is no longer able to stimulate GREM1 [21]. The result is stimulation of BMP signaling, suppression of FGF8, overexpression of retinoic acid, and finally web separation. Therefore, an abnormally high SHH activity in the limb bud will lead to persistence of GREM1 activity and syndactyly. In ducks and bats, the normally webbed digits/wings are caused by a persistent GREM1 expression [23]. In humans, gene mutations that lead to an increase in the activity of SHH will also lead to persistent GREM1 expression and syndactyly (Table 1). Point mutations/duplications within the ZRS or the surrounding region of 7q36 lead to increased SHH activity and syndactyly [8, 24, 25]. RAB23, TWIST1, and GLI3 normally act as negative regulators of SHH [26–28]. Hence, loss-of-function mutations of *RAB23*, *TWIST1*, and *GLI3* will be associated with a high SHH activity and syndactyly (Table 1 and Figure 1).

The last protein acting on Step I is the CX43 protein. Mutations of *GJA1* lead to loss of the functional activity of CX43, which in turn leads to reduced BMPs and syndactyly (Syndactyly type 3, OMIM 186100) [29].

### 3.2. Step II: Related Syndactyly in Humans

In Step II, acrocephalo-syndactyly syndromes caused by gain-of-function mutations of *FGFR1*/*FGFR2* are associated with an increased activity of FGF8 and syndactyly. Overexpression of other FGFs such as FGF2 in the cranium also occurs, leading to craniosynostosis. The severity of craniofacial abnormalities versus hand syndactyly will also depend on the type of mutation. A classic example is Apert syndrome (OMIM 101200). Hand surgeons classify patients with Apert syndrome with *FGFR2* mutations into two groups according to the severity of syndactyly/craniofacial defects [30, 31]. The first group of patients have “severely affected” heads but “mildly affected” hands and carry the mutation Ser252Trp in *FGFR2*. The resulting gain-of-function of the receptor in this mutation leads to an increased functional effect of FGF2 which is mainly expressed in the craniofacial skeleton. The second group have “mildly affected” heads but “severely affected” hands and carry the mutation Pro253Arg in *FGFR2*. The resulting gain-of-function of the receptor in this mutation results in loss of the ligand-binding specificity in which the abnormal receptor is able to bind to FGF10, which results in upregulation of ectodermal FGF8 through the FGF10–FGF8 loop [31].

The pathophysiology of the synpolydactyly type 2b (*FBLN1* mutations, OMIM 608180) also occurs at Step II. Normally, the FIBULIN1 protein binds to FGF8 with high affinity, modulating its activity and expression [32]. Hence, the pathogenesis of this type of syndactyly is through Step II.

### 3.3. Step III: Related Syndactyly in Humans

*HOXD13* mutations have been linked to two types of human syndactyly (Table 1) including the rare type 2a Vordinborg synpolydactyly (OMIM 186000) and type 5 syndactyly (OMIM 186300) [33]. Experimental models have shown that the mutated *Hoxd13* has a direct suppressive effect on retinoic acid in the autopod [34]. Hence, the pathogenesis of HOXD13-related syndactyly is through Step III (Figure 1 and Table 1). Brison et al. [35] reviewed the literature on *HOXD13* mutations and found that the associated phenotypes included various forms of brachydactyly, syndactyly, and synpolydactyly. The pathogenesis of the polydactyly component in the Vordinborg synpolydactyly (OMIM 186000) is probably related to *HOXD13*-GLI3R interactions. Chen et al. [36] have shown experimentally that *Hoxd13* directly binds to Gli3r (the repressor form of Gli3). The mutated *Hoxd13* directs Gli3r for a premature degradation. This will result in polydactyly, similar to the polydactyly phenotype caused by depletion of Gli3r. Brison et al. [35] have also brought the attention that the G11A missense mutation of the *HOXD13* gene in humans with synpolydactyly confirms a novel functional domain in *HOXD13* which regulates digit number through its interaction with GLI3R.

### 3.4. Step I: Related Animal Models of Syndactyly

Animal models of syndactyly may also be classified according to the 3-step pathway of pathogenesis (Table 2).

Mice with *Lrp4* mutations develop polysyndactyly in their forelimbs and hindlimbs [37]. The Lrp4 protein is a strong suppressor of WNT signaling, and hence, loss-of-function mutations of *Lrp4* will lead to signal activation and syndactyly [37].

The murine limb deformity (Ld) model is caused by *Fmn1* deletions and show oligosyndactyly, renal defects, and radio-ulnar synostosis [38]. The phenotype of the Ld model is considered to be the closest phenotype to the phenotype of Cenani–Lenz syndrome in humans. The phenotype of transgenic chicks with Grem1 overexpression [39] is also similar to the human syndactyly phenotype associated with duplications encompassing the *GREM1* gene [20].

Other animal models of syndactyly were created by suppression of Bmp signaling. Overexpression of the Bmp antagonist Noggin in mice resulted in extensive soft-tissue syndactyly and postaxial polydactyly [40]. Inactivation of the mouse Bmp receptor gene *Bmpr1a* in the limb bud was associated with upregulation of both Fgf8 and Fgf4, resulting in syndactyly [41]. Bmp2-deficient mice display soft-tissue syndactyly of the third web space, while combined deficiency of Bmp2 and Bmp4 in the limb bud results in complete syndactyly of all limbs [42]. Mice lacking Cx43 show a reduction in the expression of Bmp2 leading to a secondary overexpression of Fgfs and syndactyly [29]. SMADs 1 and 5 are downstream of the BMP signaling. Selective inactivation of Smads 1 and 5 in mice results in overexpression of Fgf8 and syndactyly [43].

Finally, the hammertoe (Hm) mutant mouse model shows syndactyly of digits 2–5. In this spontaneous mouse mutation, a 150 kb noncoding DNA fragment from chromosome 14 is inserted upstream of the *Shh* promotor. This results in overexpression of interdigital Shh, secondary suppression of the Bmp signaling, and finally syndactyly. The pathophysiology of this secondary suppression of Bmp was recently shown by Mouri et al. [44]. The overexpression of Shh leads to the upregulation of Chordin. Chordin binds to Bmps and sequestrates them into latent complexes, thereby suppressing the Bmp activity [44].

### 3.5. Step II: Related Animal Models of Syndactyly

Suppression of notch signaling in mice results in increased expression of Fgf8 in the AER and syndactyly [45]. Notch 1 and its ligand Jagged2 are coexpressed in the AER. Suppression of Notch signaling has been demonstrated in mice lacking *Jagged2*. This resulted in an increased expression of Fgf8 and fusion of the middle three digits. Since Notch is also expressed in the thymus and the craniofacial area, mutant mice also exhibited cleft palate, tongue fusion, and thymic defects [46].

*Msx1*/*Msx2* double-mutant mice show variable phenotypes including oligodactyly, polydactyly, and syndactyly. This is associated with extended Fgf activity in the AER [47].

The *FRAS1* gene encodes an extracellular matrix protein involved in the establishment of the epidermal basement membrane. Mutations in *FRAS1* in humans cause Fraser syndrome (OMIM 219000) with eye, kidney, and craniofacial defects [48]. Hines et al. [49] identified a novel ENU-derived rounded foot (rdf) mouse mutant with hindlimb cutaneous syndactyly caused by loss-of-function nonsense allele of *Fras1*. The primary defect in these animals was the decreased Msx2 expression [49]. As mentioned above, deficiency of Msx1/2 is associated with extended Fgf activity in the AER [47].

Basement membranes are extracellular matrices underlying the epithelium and endothelium of various organs including the AER. All basement membranes contain at least one member of Laminin, type IV collagen, and Nidogen families [50]. Two Nidogen isoforms (Nidogens 1 and 2) have been identified in vertebrates. The individual knockout of either *Nid1* or *Nid2* in mice does not affect basement membrane formation and animals show no abnormalities. Mice lacking both Nidogens have defective ectodermal basement membrane of multiple organs including aberrant AER formation, altered distribution of Fgf8, and soft-tissue syndactyly [51]. Since basement membrane formation of multiple organs are affected, perinatal lethality with multiorgan defects are also seen in these animals [50]. This model shows that altered distribution of Fgf8 may also lead to syndactyly.

### 3.6. Step III: Related Animal Models of Syndactyly

Animal models with suppressed retinoic acid activity show syndactyly [17, 18]. The retinaldehyde dehydrogenase-2 enzyme is encoded by the *Raldh2* gene. The enzyme oxidizes retinaldehyde to retinoic acid. Retinoic acid functions as a ligand for nuclear retinoic acid receptors (known as RAR) to induce the transcription of target genes. Hence, suppression of retinoic acid activity in experimental mice may be done either by targeting the retinaldehyde dehydrogenase-2 enzyme or the RAR receptors. *Raldh2*^–/–^ autopods show syndactyly secondary to suppression of Mmp11 (which is responsible for interdigital ECM degradation). In this model, Fgf8 expression is normal, indicating that retinoic acid acts downstream of Fgf8 in the pathogenesis of syndactyly [17]. *RARb*/*RARg* double-mutant mice also show syndactyly secondary to the suppression of Mmp11 [18].

Kuss et al. [34] used the naturally occurring mouse mutant (Spdh/Spdh mutant) that has the polyalanine expansions in homeobox d13 (*Hoxd13*). The authors showed that the mutated *Hoxd13* has a direct suppressive effect on retinoic acid in the autopod [34]. Intrauterine treatment with retinoic acid restored pentadactyly in Spdh/Spdh mice [34].

The proteins Bid, Bim, and Puma act to activate Bax and Bak which are essential to initiate the apoptotic pathway at the mitochondrial level. Hence, *Bid*/*Bim*/*Puma* triple knockout mice show reduced apoptosis and syndactyly. Besides Mmp11, the ADAMTS group of peptides is essential in interdigital ECM degradation. Versican (a proteoglycan) is an important component of the interdigital ECM. ADAMTS mediates the cleavage of versican. Hence, mice deficient in ADAMTS exhibit syndactyly [16].

Akirins (Akirin 1 and 2) are small nuclear proteins that localize to promoter and enhancer regions of genes. They function as “bridge” proteins to coordinate gene expression patterns [52]. *Akirin2* null embryos are not able to survive beyond embryonic day 9.5. Transgenic mouse models with knockout of *Akirin2* in the limb epithelium leads to a loss of interdigital cell death and an increase in cell proliferation, resulting in the retention of the interdigital web and soft-tissue syndactyly [52].

## 4. Discussion

Limb development is a complex process involving the action of signaling centers that coordinate spatially and temporally to sculpt a limb [22]. Digit formation requires the combined coordination of morphogen gradients and feedback loops that dictate responses by cells of the AER, zone of polarizing activity in which SHH is expressed, nonAER ectoderm, and mesenchymal cells within the limb bud. Finally, the regulation of interdigital tissue regression also requires the interplay of multiple spatiotemporally controlled morphogen gradients to ensure proper limb formation and release of individual digits. Understandably, syndactyly may originate when there is a failure in the regulation of interdigital tissue regression. The limb morphogenesis is better understood when the genetic networks are categorized with respect to well-established proximodistal, anteroposterior, and dorsoventral axes [22]. FGF8, SHH, and WNT7A/EN1 are the main controllers of these 3 axes, respectively. Congenital limb defects may be classified according to the axis defect. For example, brachydactyly and amelia are related to defects in the proximodistal axis; polydactyly as well as radial/ulnar ray deficiencies are related to defects in the anteroposterior axis; and ventral/dorsal dimelia (the appearance of palmar structures on the dorsal aspect of the hand and the appearance of dorsal structures on the ventral aspect of the hand, respectively) are related to defects in the dorsoventral axis [53, 54]. Hence, it would be pertinent to understand the origin of syndactyly with respect to the developmental axis. Oberg [53] studied this extensively and concluded that syndactyly is best classified under malformations of “unspecified” axis [53]. However, since FGF8 is the key middle step mediating the pathogenesis of syndactyly, one may argue that syndactyly may be considered as a defect in the proximodistal axis (FGF8 is the main controller of this axis).

The current review offers a unified pathway that could explain the pathogenesis of syndactyly in humans and experimental animals. The pathway is a 3-step pathway, and hence, human and animal syndactyly may be classified accordingly (Tables 1 and 2). The final step in the pathway is the suppression of retinoic acid in the mesenchyme which will lead to the suppression of both apoptosis and ECM degradation. It is important to realize that retinoic acid only induces apoptosis/EMC degradation within the interdigital space and not within the digits. The explanation for this was shown by Zhao et al. [17]. The digits (and not the interdigital spaces) express a cytochrome known as Cyp26b1 which inactivates retinoic acid. Hence, mesodermal retinoic acid is unable to “degrade” the digits.

The pathway proposed in the current review explains the pathogenesis of most types/models of syndactyly. Certain types of syndactyly have a different pathway of pathogenesis (such as Poland syndrome syndactyly) or the pathogenesis is yet to be determined (such as Shaker syndactyly mouse models, syndactyly related to b-HLHA9 in both humans and animals, syndactyly of *Noggin*-null mice, syndactyly of *Sp6* mutant mice, and isolated 4/5 metacarpal fusion in humans). These types of syndactyly are summarized in Table 3.

Poland syndrome (OMIM 173800) is characterized by the unilateral absence or hypoplasia of the sternocostal head of the pectoralis major muscle and ipsilateral symbrachydactyly. However, several other pectoral muscle and hand defects have also been described [55]. One characteristic radiological feature in the hands of these patients is the pronounced hypoplasia or aplasia of the middle phalanx [56]. Poland syndrome results from a vascular insult to the subclavian artery during the 19th embryonic stage. During this stage, three events normally occur: the development of the sternocostal head of the pectoralis major, the chondrification of the middle phalanges and the initial phase of finger separation [57]. Hence, the pathogenesis of syndactyly in Poland syndrome is not related to our 3-step pathway.

The mouse mutant Shaker-with-syndactylism (Sy) is caused by *Fibrillin2* (*Fbn2*) loss-of-function mutations [58]. Fibrillin2 is an important component of elastic fibers and the pathophysiology of “Sy” syndactyly is unknown. However, Fibrillins are known to interact with Fiblulins [59]. Since FIBULIN1 deficiency leads to increased FGF8 activity and syndactyly (see Table 1), these interactions may explain the pathogenesis of syndactyly in Shaker mice.

The Basic Helix-Loop-Helix Member A9 (b-HLHA9, also known as FINGERIN) is a transcription factor which is expressed in the distal hand/foot plates [60]. Duplications of *BHLHA9* result in split hand-foot malformation with long-bone deficiency type 3 (SHFLD 3, OMIM 612576) [61]. In contrast, loss-of-function mutations of *BHLHA9* lead to mesoaxial synostotic syndactyly with phalangeal reduction (MSSD, Type 9 human syndactyly, OMIM 609432) [3]. Schatz et al. [60] have established that complete deletion of the murine ortholog of *Fingerin* causes syndactyly, both in vivo and ex vivo. However, the exact pathogenesis of syndactyly is still unknown, and two different theories have been proposed [60]. The first theory is based on the fact that b-HLHA9 is a down-stream target of Notch signaling [62]. As mentioned above (see Table 2), suppression of Notch signaling leads to an increased expression of Fgf8 in the AER and syndactyly [45]. The second theory is based on the fact that b-HLHA9 contains a unique proline-rich carboxy-terminus not typically found in other bHLH factors [60]. Proline-rich domains participate in protein-protein interactions which may sequester the function of proteins that provide the apoptotic signal [60].

Mutations in *NOGGIN* in humans leads to various phenotypes including brachydactyly type B2 (OMIM 611377) and various forms of synostosis/ankylosis/symphalangism (OMIM 186500, 184460, 185800, 186570). In contrast, cutaneous syndactyly is a prominent feature in *Noggin*-null mice [63]. The exact pathogenesis of syndactyly is unknown but is thought to be related to the secondary overexpression of Indian hedgehog (Ihh) in the interdigital spaces, which in turn leads to reduced apoptosis [63].

Sp6 (also known as Epiporfin) and Sp8 (also known as Buttonhead) are transcription factors implicated in AER induction and maintenance [64]. They are expressed in the limb bud ectoderm and AER and induce the expression of Fgf8 in the AER [64]. *Sp8* mutant mice exhibit severe loss of Fgf8 expression in the AER leading to limb truncations. In contrast, *Sp6* mutant mice show a mild loss of Fgf8 expression and a mild syndactyly phenotype [64]. The pathogenesis may be explained by the fact that animal models with mild deficiency of Fgf8 in the AER show secondary overexpression of Fgf4 (normally, Fgf4 is restricted to the posterior AER) [65]. As a result of this ectopic Fgf4 expression, all of the skeletal defects caused by the loss of Fgf8 are rescued, and syndactyly is seen in the phenotype of experimental animals [66]. This pathogenesis also explains the occasional presence of syndactyly instead of ectrodactyly in one of the limbs in patients with split hand-foot malformations (which are associated with suppression of FGF8 in the AER) [67, 68].

Isolated fusion of the 4/5 metacarpal has been classified as syndactyly type 8 (OMIM 309630). This is the only type of syndactyly that is inherited as X-linked and is caused by nonsense mutations in *FGF16* [69]. The pathogenesis of the isolated metacarpal fusion is unknown. However, *Fgf16* knockdown in experimental animals is known to affect Fgf8 expression in the AER as well as the expression of Shh expression in the zone of polarizing activity [70].

Our review also demonstrates that mutations in humans and animals do not always result in similar phenotypes. *Fbn2* mutations in mice show syndactyly, while *FBN2* mutations in humans are associated with congenital contractual arachnodactyly (OMIM 121050). Another example is the mouse model of Apert syndrome which showed most of the classic craniofacial features, but none of the animals exhibited syndactyly [71]. A third example is the phenotype associated with the loss of SMADs (SMADs 1/5/8-SMAD 4 complexes act as transcription factors for the BMP signaling pathway). In animals, deficiency of Smads 1/5 results in a syndactyly phenotype [43]. In humans, no specific syndromes are related to SMAD 1/5 deficiency. Instead, loss-of-function mutations of SMAD 4 in humans are associated with Juvenile polyposis syndromes (OMIM: 174900 &175050).

Another observation from the current review is the correlation between the degree of pathway abnormality and the severity of syndactyly phenotype. One example is seen in Apert patients. The “severely affected” hand group has a more severe syndactyly phenotype and higher FGF8 overactivity when compared to the “mildly affected” hand group. Another example is Cenani–Lenz syndrome (OMIM 212780) caused by *LRP4* mutations. The classic syndrome is characterized by bilateral hand and feet syndactyly along with metacarpal/metatarsal synostosis and phalangeal disorganization. Hand function in these patients is poor even after surgical correction [72]. This relatively severe classic syndactyly phenotype is associated with homozygous and compound heterozygous missense and splice mutations of the *LRP4* gene [19]. In contrast, missense mutations of the *LRP4* gene cause a very mild phenotype with simple syndactyly and well-developed digits, without metacarpal synostosis/phalangeal disorganization [73]. Furthermore, complete loss-of-function mutations of *LRP4* lead to the most severe oligosyndactyly phenotype and may even be lethal with intrauterine demise [74–76]. A third example is seen in Type 2a human synpolydactyly (OMIM 186000). A mild syndactyly phenotype is seen with heterozygous *HOXD13* mutations, and a severe syndactyly phenotype is seen with homozygous *HOXD13* mutations [33, 77].

Finally, the author noted that the syndactyly phenotype related to *LRP4* and *APC* mutations (which directly act on the WNT canonical pathway in Step I) and those directly leading to persistence of GREM1 expression (which directly act on the BMP canonical pathway in Step I) will have a similar Cenani–Lenz phenotype. Hence, syndactyly related to mutations of *LRP4*, *APC*, *FMN1*, and *GREM1* should be grouped together. In contrast, human syndactyly related to high SHH signaling has a different syndactyly phenotype and is associated with preaxial polydactyly, although the pathogenesis is still at Step I (Figure 1). This may be explained by the fact that the high SHH is acting indirectly on GREM1 (via the epithelial-mesenchymal feedback loop). Furthermore, the high SHH expression is expected to result in ectopic anterior expression of SHH, leading to concurrent preaxial polydactyly [78]. This explains the phenotype of syndactyly and concurrent polydactyly in Haas (OMIM 186200), triphalangeal thumb polysyndactyly (OMIM 174500), Carpenter (201000), Saethre-Chotzen (OMIM 101400), and Greig (OMIM 175700) syndromes (Table 1).

## 5. Conclusions and Future Perspectives

The 3-step pathway of pathogenesis provides a novel look at the correlation between the genetics and pathogenesis of syndactyly. The syndactyly is best classified according to the pathogenesis since the phenotype is affected by the site along the pathway. The pathway may also help to guide research on syndactyly types with undetermined pathogenesis. For example, the mouse mutant Shaker-with-syndactylism (Sy) is caused by *Fibrillin2* (*Fbn2*) loss-of-function mutations [58]. Since Fibrillins and Fibulins are both important components of elastic fibers and both are known to interact [59], the pathogenesis of Sy syndactyly may be investigated to determine if it is through Fibulin1 as a modifier of Fgf8 activity in Step II.

## Figures and Tables

**Table 1:** Syndactyly in humans classified according to the steps shown in Figure 1.

Step affected	Gene mutation	Name of syndrome or type of syndactyly as per Malik's classification (OMIM if available)
IA: activation of the WNT canonical signaling or the accumulation of beta catenin	(i) *LRP4*	(i) Cenani–Lenz syndrome or type 7a syndactyly (212780)
	(ii) *APC*	(ii) Cenani–Lenz phenotype

IB: suppression of the BMP canonical signaling	(i) *FMN1* deletions or duplication encompassing *GREM1*-*FMN1*	(i) Cenani–Lenz phenotype or type 7b syndactyly
	(ii) *ZRS*	(ii) Haas (type 4) syndactyly (186200); triphalangeal thumb polysyndactyly syndrome (174500)
	(iii) *RAB23*	(iii) Carpenter syndrome (201000)
	(iv) *TWIST1*	(iv) Saethre–Chotzen syndrome (101400)
	(v) *GLI3*	(v) Greig syndrome (175700) and other GLI3-related syndactyly
	(vi) *GJA1*	(vi) Johnston–Kirby type 3 syndactyly (186100)

II: increased activity of FGF8	(i) Gain-of-function mutations of *FGFR1* or *FGFR2*	(i) Pfeiffer (101600), Apert (101200), and Saethre-Chotzen (101400) syndromes.
	(ii) *FBLN1*	(ii) Debeer type 2b syndactyly (608180)

III: suppression of retinoic acid or suppression of apoptosis/matrix degradation	(i) *HOXD13*	(i) Vordingborg type 2a syndactyly (186000) and syndactyly type 5 (186300)

**Table 2:** Syndactyly in animal models classified according to the steps shown in Figure 1.

Step affected	Animal model of syndactyly
IA: activation of the WNT canonical signaling or the accumulation of beta catenin	Mice with *Lrp4* mutations

IB: suppression of the BMP canonical signaling or the suppression of BMPs/SMADs	(i) Murine limb deformity (Ld) model (*Fmn1* deletions resulting in Grem1 overexpression)
	(ii) Transgenic chick with Grem1 overexpression
	(iii) Overexpression of the Bmp antagonist Noggin
	(iv) Inactivation of the Bmp receptor gene *Bmpr1a*
	(v) Mice deficient in Bmp2 and Bmp4
	(vi) Knockout mice lacking Cx43
	(vii) Selective inactivation of Smad 1 and 5.
	(viii) Hammer toe (Hm) mutant mice.

II: overexpression of FGF8	(i) Suppression of Notch signaling
	(ii) *Msx1*; *Msx2* double-mutant mice
	(iii) Rounded foot mouse mutants (*Fras1* loss-of-function)
	(iv) Mice lacking Nidogen 1 and 2.

III: suppression of retinoic acid or suppression of apoptosis/extracellular matrix degradation	(i) Suppression of retinoic acid activity
	(ii) Spdh/Spdh mice
	(iii) *Bid*, *Bim*, *Puma* triple knockout mice
	(iv) Mice deficient in ADAMTS
	(v) Knockout of *Akirin2* in the limb ectoderm

**Table 3:** Other types of syndactyly with a unique pathway of pathogenesis or with an undetermined pathogenesis.

Type of syndactyly	Animals or humans	Gene mutation/knockout	Proposed pathogenesis as per our literature review
Poland syndrome (OMIM 173800)	Humans	—	Unique pathway: vascular insult to the subclavian artery during the 19^th^ embryonic stage
Shaker syndactyly	Mice	*Fbn2*	Fibrillin-Fibulin interactions leading to increased Fgf8 activity (step II)
Type 9 human syndactyly (OMIM 609432) and mice deficient in BhLha9	Humans/mice	Loss-of-function mutations of *BHLHA9* and deletion of the murine ortholog *Bhlha9*	Either through suppression of Notch signaling (step II, see Table 2) or disturbance of the apoptotic signal (step III)
*Noggin*-null mice	Mice	*Nog*	The overexpression of Ihh in the interdigital spaces leading to reduced apoptosis (step III)
*Sp6* mutant mice	Mice	*Sp6*	The mild deficiency in Fgf8 leads to overexpression of Fgf4 in the AER resulting in syndactyly
Type 8 human syndactyly (OMIM 309630)	Humans	*FGF16*	Either via altering the expression of FGF8 or SHH

AER: apical ectodermal ridge.
